# *Leishmania infantum* Amastigotes Enhance HIV-1 Production in Cocultures of Human Dendritic Cells and CD4^+^ T Cells by Inducing Secretion of IL-6 and TNF-α

**DOI:** 10.1371/journal.pntd.0000441

**Published:** 2009-05-26

**Authors:** Ravendra Garg, Corinne Barat, Michel Ouellet, Robert Lodge, Michel J. Tremblay

**Affiliations:** Centre de Recherche en Infectiologie, Centre Hospitalier de l'Université Laval, and Faculté de Médecine, Université Laval, Québec, Québec, Canada; Fundação Oswaldo Cruz, Brazil

## Abstract

**Background:**

Visceral leishmaniasis has emerged as an important opportunistic disease among patients infected with HIV-1. Both HIV-1 and the protozoan parasite *Leishmania* can productively infect cells of the macrophage-dendritic cell lineage.

**Methodology/Principal Findings:**

Here we demonstrate that *Leishmania infantum* amastigotes increase HIV-1 production when human primary dendritic cells (DCs) are cocultured together with autologous CD4^+^ T cells. Interestingly, the promastigote form of the parasite does not modulate virus replication. Moreover, we report that amastigotes promote virus replication in both cell types. Our results indicate that this process is due to secretion of parasite-induced soluble factors by DCs. Luminex micro-beads array system analyses indicate that *Leishmania infantum* amastigotes induce a higher secretion of several cytokines (i.e. IL-1α, IL-2, IL-6, IL-10 and TNF-α) and chemokines (i.e. MIP-1α, MIP-1β and RANTES) in these cells. Studies conducted with pentoxifylline and neutralizing antibodies revealed that the *Leishmania*-dependent augmentation in HIV-1 replication is due to a higher secretion of IL-6 and TNF-α.

**Conclusions/Significance:**

Altogether these findings suggest that the presence of *Leishmania* within DC/T-cell conjugates leads to an enhancement of virus production and demonstrate that HIV-1 and *Leishmania* can establish complex interactions in such a cellular microenvironment.

## Author Summary

Visceral leishmaniasis (VL) is a potentially deadly parasitic disease afflicting millions worldwide. Although itself an important infectious illness, VL has also emerged as an opportunistic disease among patients infected with HIV-1. This is partly due to the increasing overlap between urban regions of high HIV-1 transmission and areas where *Leishmania* is endemic. Furthermore, VL increases the development and clinical progression of AIDS-related diseases. Conversely, HIV-1-infected individuals are at greater risk of developing VL or suffering relapse. Finally, HIV-1 and *Leishmania* can both productively infect cells of the macrophage-dendritic cell lineage, resulting in a cumulative deficiency of the immune response. We therefore studied the effect of *Leishmania infantum* on HIV-1 production when dendritic cells (DCs) are cocultured with autologous CD4^+^ T cells. We show that amastigotes promote virus replication in both DCs and lymphocytes, due to a parasite-mediated production of soluble factors by DCs. Micro-beads array analyses indicate that *Leishmania infantum* amastigotes infection induces a higher secretion of several cytokines in these cells, and use of specific neutralizing antibodies revealed that the *Leishmania*-induced increase in HIV-1 replication is due to IL-6 and TNF-α. These findings suggest that *Leishmania*'s presence within DC/T-cell conjugates leads to an enhanced HIV-1 production.

## Introduction

Leishmaniasis is today recognized as one of the world's most important parasitic diseases, threatening 350 million people in 88 countries [1]. At least 20 *Leishmania* species can induce various clinical manifestations better known as leishmaniasis. Infection by these trypanosomatid protozoan parasites may lead to a variety of symptoms, ranging from simple, self-healing skin ulcers to a severe, life-threatening visceral disease caused by the *Leishmania donovani* complex, including *Leishmania infantum*. One of the most neglected diseases, visceral leishmaniasis (VL) is the most severe form of leishmaniasis, characterized by fevers, suppressed immunity and hepatosplenomegaly.

Of particular interest, VL has also emerged as an important opportunistic infection in individuals also infected with human immunodeficiency virus type-1 (HIV-1). Indeed, the outbreak of the HIV-1/AIDS pandemic during the past two decades has dramatically modified the natural history of infection by *Leishmania* in co-infected patients. According to the World Health Organization (WHO), HIV-1/*Leishmania* co-infection has been reported in 34 countries in several regions of the globe such as Africa, Asia, Europe, and South America. The increase in the number of cases of co-infection arises from the overlap between regions of active HIV-1 transmission, mostly cities and urbanized areas, and the regions in which *Leishmania* is endemic. VL is prevalent in HIV-1/*Leishmania* co-infected individuals and is known to promote not only the development of AIDS-defining illness conditions but also its clinical progression, therefore diminishing the life expectancy of HIV-1-infected subjects. For example, in dually infected patients from southern Europe, all of the isolates were *Leishmania infantum* [1],[2]. Furthermore, HIV-1 infection increases the risk of developing VL in endemic areas, enhances the probability of relapse and is also associated with a poorer anti-parasitic drug response [1],[2]. It has been proposed that HIV-1 and *Leishmania* act in a deadly synergy. HIV-1/*Leishmania* co-infection exerts cumulative deficiency of the cellular immune response since both agents harm similar immune resources, such as macrophages and dendritic cells, for their reciprocal benefit [3]–[5].

Dendritic cells (DCs) are versatile antigen-presenting cells, recognizing and capturing invading pathogens and establishing a vital bridge between innate and adaptive immunity. DCs also play a key role in HIV-1 infection and dissemination. Indeed, it has been reported that HIV-1 is efficiently transferred from immature DCs to CD4^+^ T cells via different routes [6]–[9]. For example, after the cells capture and bind HIV-1, a rapid transfer (i.e. early transfer or *trans* infection) occurs when the virus on the surface of immature DCs or located within endosomal compartments is transported to the DC/T-cell synapse and transmitted directly to CD4^+^ T cells. This event is followed by a second phase (i.e. late transfer or *cis* infection) that is dependent on productive virus infection of immature DCs and on the eventual transfer of progeny viruses to CD4^+^ T cells. It has been proposed that after virus capture or uptake, immature DCs (iDCs) located in submucosal tissues migrate to lymphoid tissues and become mature DCs (mDCs) [10]–[12], which can potently present nominal antigens to CD4^+^ T cells in lymphoid tissues.

Although the possible multifaceted interactions between HIV-1 and *Leishmania* have been studied in macrophages [13]–[15], there is still very little information on the consequences of infection of DCs with these two microorganisms. In macrophages, *Leishmania* increases both HIV-1 gene transcription and release of progeny virus through production of proinflammatory cytokines. On the other hand, some studies have suggested that DCs can be infected by either *Leishmania* promastigotes or amastigotes [16],[17]. It has also been reported that competition between HIV-1 and *Leishmania* amastigotes for DC-SIGN binding has an impact on the HIV-1 life cycle when DCs were first exposed to the parasite before inoculation with HIV-1 [18].

Proinflammatory cytokines play an important role in both innate and adaptive immune responses against viruses and intracellular pathogens. Interestingly, TNF-α has been reported to stimulate HIV-1 replication in the promonocytic U1 cell line through NF-κB activation and subsequent trans-activation of the viral regulatory elements [19]. In addition, IL-6 has been reported to stimulate HIV-1 replication in macrophages again via a NF-κB-mediated signal transduction pathway [20]–[22]. An increased production of IL-6 and TNF-α has been detected in *Leishmania*-infected macrophages [23],[24]. Thus, in the case of HIV-1/*Leishmania* co-infected patients, it is likely that HIV-1 uses a part of the *Leishmania*-mediated cytokine network to its own advantage.

In the present work, we investigated the influence of *Leishmania* infection on the biology of HIV-1 in physiologically relevant human DCs. We demonstrate here for the first time that *Leishmania infantum* amastigotes promote HIV-1 replication in both DCs and autologous CD4^+^ T cells when these two cell subpopulations are cultured together. The mechanism responsible for this up-regulatory effect is linked with secretion of parasite-induced soluble factors by DCs.

## Materials and Methods

### Ethics statement

Human primary DCs were generated from monocytes obtained from the blood of healthy donors, in accordance with the guidelines of the Bioethics Committee of the Centre Hospitalier de l'Université Laval Research Center. A written consent was obtained from all blood donors.

### Reagents

Recombinant human interleukin-2 (rhIL-2), efavirenz (EFV) and azidothymidine (AZT) were obtained from the NIH AIDS Repository Reagent Program (Germantown, MD). Interferon-gamma (IFN-γ) and IL-4 were purchased from R&D Systems (Minneapolis, MN), whereas granulocyte macrophage–colony stimulating factor (GM-CSF) was a generous gift from Cangene (Winnipeg, MB). Lipopolysaccharide (LPS) and phytohemagglutinin-L (PHA-L) were purchased from Sigma (St-Louis, MO). The culture medium consisted of RPMI-1640 supplemented with 10% foetal bovine serum (FBS), penicillin G (100 U/mL), streptomycin (100 U/mL) and glutamine (2 mM), which were all purchased from Wisent (St-Bruno, QC), and primocine (Amaxa Biosystems, Gaithersburg, MD).

### Isolation and culture of cells

In brief, CD14^+^ cells (i.e. monocytes) were isolated from peripheral blood mononuclear cells, using a monocyte-positive selection kit according to the manufacturer's instructions (CD14-positive selection kit; StemCell Technologies Inc., Vancouver, BC) and cultured in six-well plates (10^6^ cells/ml). Immature DCs (iDCs) were generated from these purified monocytes by a treatment with GM-CSF (1,000 U/ml) and IL-4 (200 U/ml) for 7 days. The maturation of iDCs was induced on the fifth day by culturing them for 48 h with the above-described cytokines supplemented with IFN-γ (1,000 U/ml) and LPS (100 ng/ml). The final phenotype of iDCs and mature monocyte-derived DCs (mDCs) was monitored by flow cytometry (data not shown). For example, iDCs expressed HLA-DR, CD86, DC-SIGN, CD1a and low levels of CD14, whereas mDCs expressed CD83 and high levels of ICAM-1, HLA-DR, and CD86 but lower levels of DC-SIGN and CD14 compared to iDCs (data not shown). iDCs were considered effectively differentiated based on the loss of CD14 and acquisition of CD1a and DC-SIGN (data not shown). Autologous CD4^+^ T cells were isolated with a negative selection kit (StemCell Technologies) and activated (2×10^6^ cells/ml) with the mitogenic agent PHA-L (1 µg/ml) and rhIL-2 (30 U/ml) for 48 h prior to their use.

### Viral preparations

Viruses were produced upon transient calcium phosphate transfection of 293T cells either with pNL4-3Bal*env*, an R5-tropic infectious molecular clone of HIV-1 [25], or pNLBalHSA-IRES (see below for more details). Virus stocks were normalized for virion content by using a sensitive in-house, double-antibody sandwich enzyme-linked immunosorbent assay specific for the major viral core p24 protein [26]. Viral preparations underwent a single freeze-thaw cycle before being used in subsequent experiments. The pNLBalHSA-IRES molecular construct has been described previously [27] and was obtained by replacing the *eGFP* gene in the NLENG1-IRES vector (NL4-3 backbone) with the coding sequence for mouse heat stable antigen (HSA) and replacing the X4-tropic *env* gene of NL4-3 with that of the R5-using *env* gene of NL4-3Bal*env*.

### Culture of *Leishmania infantum* parasites

The *Leishmania infantum* strain MHOM/MA/67/ITMAP-263 was maintained at 27°C by a weekly passage in RPMI-1640 supplemented with 10% FBS, buffered with 25 mM HEPES and 2 mM NaHCO_3_, containing 5 µg/ml haemin and antibiotics. Axenic *Leishmania infantum* amastigotes were differentiated *in vitro* from stationary-phase promastigotes. The culture and maintenance of axenic amastigotes have been described previously [28]. These amastigotes showed morphological, biochemical and biological characteristics similar to those of amastigotes isolated *in vivo* [28].

### Virus transmission assays

DCs (both iDCs and mDCs) were first pulsed with NL4-3Bal*env* (10 ng of p24/10^5^ cells) for 60 min at 37°C and unbound virus was eliminated by extensive washes with phosphate-buffered saline (PBS). Next, DCs were exposed to *Leishmania infantum* promastigotes or axenic amastigotes at a parasite/cell ratio of 10∶1 for up to 4 h and free parasites were washed out with warm PBS. Cells were then incubated with activated CD4^+^ T cells at a 1∶3 ratio (DCs∶CD4^+^ T cells). Viral production was assayed by measuring the cell-free p24 content at different time intervals. In some experiments, DCs were treated either with EFV (50 nM) or AZT (10 µM) for 30 min before pulsing with virions.

In virus transfer experiments aimed at evaluating the percentage of virus-infected CD4^+^ T cells, we used NLBalHSA-IRES virus. Three days after initiation of the coculture, cells were stained either with an isotype-matched irrelevant control antibody or anti-CD3 (American Type Culture Collection, Manassas, VA) followed by an FITC-conjugated goat anti-mouse IgG (Jackson ImmunoResearch Laboratories, West Grove, PA) and biotin-conjugated anti-HSA followed by R-PE-labelled streptavidin (the latter two from BD Pharmingen, San Diego, CA). Stained cells were fixed with 2% paraformaldehyde for 30 min at 4°C and then analyzed by flow cytometry (Epics ELITE ESP; Coulter Electronics, Burlington, VA).

In some HIV-1 transfer studies, permeable cell inserts with polycarbonate membranes (Transwell, Corning Inc., Lowell, MA) (pore size: 1 µm) were used to separate DCs and CD4^+^ T cells. iDCs were exposed (or not) to parasites for 3 h, washed with PBS and transferred into a culture plate. Next, activated autologous CD4^+^ T cells were pulsed with HIV-1 for 2 h, washed with PBS and either cocultured directly with iDCs or transferred into permeable cell inserts and cocultured with iDCs for 3 days. Virus production was assessed by estimating the p24 content.

### Acute virus infection of iDCs and CD4^+^ T cells

*De novo* virus production in iDCs was monitored by incubating the cells for 1 h at 37°C with NL4-3Bal*env* (10 ng of p24/10^5^ cells). After three washes with PBS, the cells were maintained in complete culture medium without autologous CD4^+^ T cells. Virus production was estimated by quantifying the p24 content in cell-free culture supernatants. In some studies, autologous activated CD4^+^ T cells were inoculated with NL4-3Bal*env* for 2 h and excess virus washed off with PBS. Next, iDCs were either left untreated or exposed to *Leishmania infantum* amastigotes for 3 h, washed, and cocultured with HIV-1-exposed autologous CD4^+^ T cells at a 1∶3 ratio. Virus production was determined by measuring p24 levels in the culture supernatants.

To analyze the effect of DC-derived soluble factors on viral production in CD4^+^ T cells, 0.22 µm-filtered culture supernatants (Millipore, Billerica, MA) were used in parallel experiments. Supernatants were harvested from unstimulated (control) or amastigote-pulsed iDCs after 24 h of culture. Next, activated autologous CD4^+^ T cells were inoculated with NL4-3 Bal*env* for 2 h and excess virus was washed off with PBS. Infected CD4^+^ T cells were then incubated for 3 days with filtered supernatants from DCs.

### Cytokine multiplex assay and blocking studies

A commercial multiplex cytokine/chemokine assay that can detect and quantify different cytokines and chemokines (i.e. IL-1α, IL-2, IL-4, IL-6, IL-10, IL-12, IL-15, TNF-α, IFN-γ, MIP-1α, MIP-1β and RANTES) through the use of the Luminex 100 apparatus was purchased from Bio-Rad (Wilmington, DE). The Luminex technology is a bead array cytometric analyzer designed to study numerous analytes simultaneously by using spectrally distinct beads in a single well of a microtiter plate, using very small sample volumes (i.e. as little as 25 µl). Briefly, iDCs were either left unexposed or exposed to *Leishmania infantum* amastigotes for 4 h and free parasites were removed with warm PBS. Cell-free supernatants were harvested from unstimulated (control) or *Leishmania*-exposed iDCs after 24 h of culture. Quantification was achieved by measuring concentrations of the studied cytokines/chemokines in cell-free supernatants according to the manufacturer's instructions (Bio-Rad).

The TNF-α inhibitor pentoxifylline (PTX) (1-(5′-oxohexyl)-3,7-dimethylxanthine; Sigma-Aldrich; 100 µM final concentration), neutralizing anti-TNF-α (R&D Systems; 500 ng/ml final concentration) and neutralizing anti-IL-6 (R&D Systems; 10 µg/ml final concentration) were added to the culture medium when initiating virus transmission or during acute infection studies and then every 2 or 3 days with each medium change.

### Statistical analysis

The statistical significance of the results was defined by performing a one-way ANOVA analysis of variance with Dunnett's post-tests to compare treated versus untreated control samples. All analyses were performed on raw data (i.e. p24 concentrations). P values lower than 0.05 were considered statistically significant. InStat software (version 3.05; GraphPad Software) was used for all analyses.

## Results

### *Leishmania* amastigotes enhances HIV-1 replication in cocultures consisting of DCs and CD4^+^ T cells

**Figure 1:**
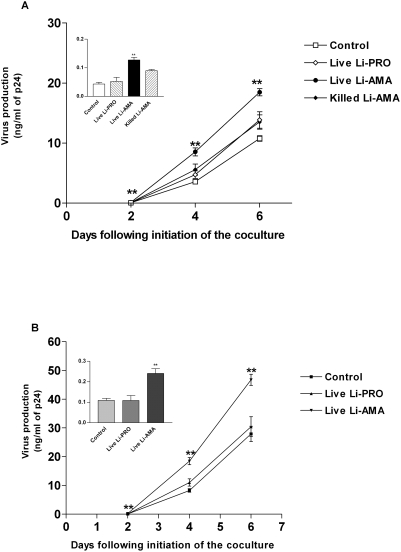
*Leishmania infantum* amastigotes enhance HIV-1 replication in cocultures consisting of DCs and autologous CD4^+^ T cells. First, iDCs (A) and mDCs (B) were pulsed for 60 min with NL4-3Bal*env* and either left unexposed (Control) or exposed to live *Leishmania infantum* promastigotes (Live Li-PRO), live *Leishmania infantum* amastigotes (Live Li-AMA), or killed *Leishmania infantum* amastigotes (Killed Li-AMA). Next, cells were cocultured with autologous CD4^+^ T cells and virus production was estimated by measuring p24 levels in the culture supernatants at the indicated time points. Virus production at day 2 following initiation of the coculture is depicted in the small inserts (upper left part of each panel). Data shown represent the means±SEM of triplicate samples and are representative of six independent experiments. Asterisks denote statistically significant differences from the cells infected with HIV-1 only (**, P<0.01).

In an attempt to provide precious information on possible relationships between *Leishmania* and HIV-1, we studied the effect of *Leishmania infantum* on HIV-1 replication in the context of cocultures constituted of DCs and autologous CD4^+^ T cells. To this end, both iDCs and mDCs were first pulsed with R5-tropic virus and next exposed to *Leishmania infantum* amastigotes and promastigotes. Thereafter, DCs were cocultured with autologous CD4^+^ T cells. A statistically significant enhancement of virus replication was observed when iDCs were exposed to the amastigote form of the parasite (Fig. 1A). Similar observations were made when coculture experiments were performed with mDCs (Fig. 1B). Since promastigotes and killed amastigotes have not elicited any significant response in our experimental setup, all further experiments were performed only with the most pathologically-relevant stage of the *Leishmania* parasite in the context of HIV-1 infection, i.e. live amastigotes. The successful infection of DCs with parasites was monitored using *Leishmania infantum*-based reporter amastigotes as we described previously [15]. Laser-scanning confocal microscopy was used to detect infection with GFP-encoding parasites while a luminometer was used to measure infection with luciferase-encoding amastigotes (data not shown).

### *Leishmania* amastigotes increase virus production in both DCs and CD4^+^ T cells

**Figure 2:**
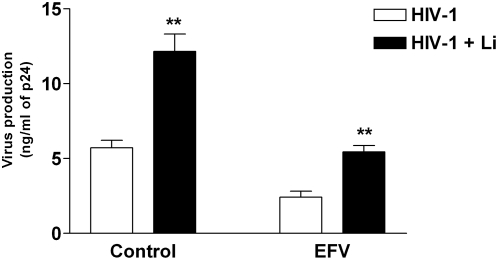
Virus replication is increased in both DCs and CD4^+^ T cells by *Leishmania infantum* amastigotes.

To define the mechanism(s) by which the parasite can promote HIV-1 replication in cocultures, iDCs were treated with the antiretroviral drug EFV during the virus pulsing period. This treatment will abrogate *de novo* virus production in iDCS without affecting transfer of viruses located on their surface or within their endosomal apparatus and the subsequent replication in CD4^+^ T cells. As expected, treatment with EFV reduces HIV-1 replication in cocultured cells (Fig. 2) and completely abolished virus production in iDCs cultured alone (data not shown). However, the parasite-mediated increase in HIV-1 transmission was detected in untreated and EFV-treated cells, therefore suggesting that *Leishmania* amastigotes are promoting virus production in both iDCs and CD4^+^ T cells. Similar observations were made when experiments were carried out with AZT (data not shown), which is another potent reverse transcriptase inhibitor.

**Figure 3:**
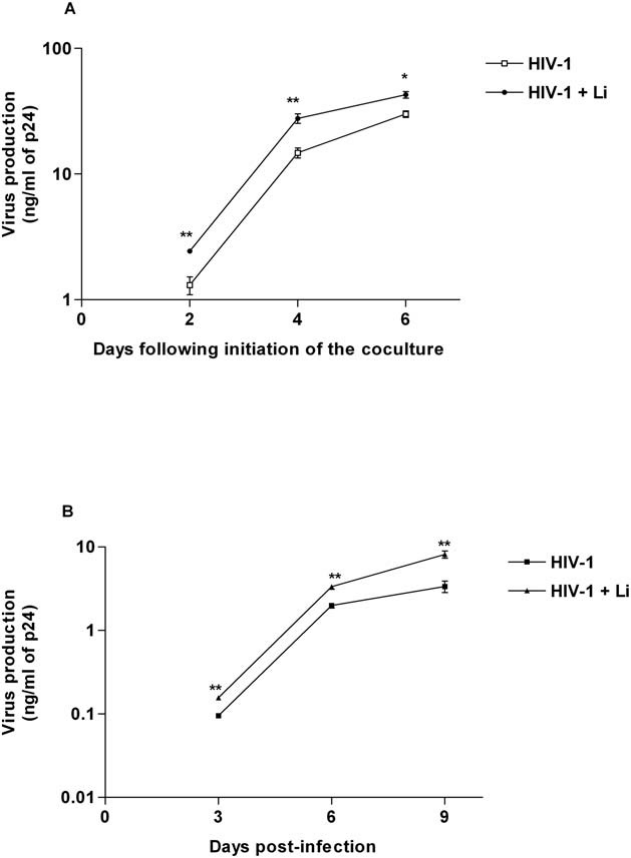
*Leishmania infantum* amastigotes enhance de novo virus production in DCs. (A) First, iDCs were pulsed for 60 min with NL4-3Bal*env* and either left unexposed or exposed to *Leishmania infantum* amastigotes (Li). Next, cells were maintained in culture for 3 days before being cocultured with autologous CD4^+^ T cells. Virus production was estimated by measuring p24 levels in the culture supernatants at 4 days following initiation of the coculture. Data shown represent the means±SEM of triplicate samples and are representative of five independent experiments. Asterisks denote statistically significant differences from the cells infected with HIV-1 only (**, P<0.01). (B) First, iDCs were pulsed for 60 min with NL4-3Bal*env* and either left unexposed or exposed to *Leishmania infantum* amastigotes (Li). Next, iDCs were maintained in culture in absence of autologous CD4^+^ T cells. Virus production was estimated by measuring p24 levels in the culture supernatants at the indicated time periods (i.e. 3, 6 and 9 days post-infection). Data shown represent the means±SEM of triplicate samples and are representative of five independent experiments. Asterisks denote statistically significant differences from the cells infected with HIV-1 only (**, P<0.01).

Next, to confirm that the parasite is affecting *de novo* virus production in iDCs, these cells were first exposed to HIV-1 and next to *Leishmania* promastigotes, and maintained in culture for 3 days before addition of autologous CD4^+^ T cells. The rationale for this experimental setup is based on the previous report that endosome-associated virions in DCs remain infectious for no more than 2 days [9]. An increase in HIV-1 transfer was still seen in the case of *Leishmania*-treated samples (Fig. 3A). To substantiate what seems to be a more productive HIV-1 infection of iDCs in the presence of *Leishmania* amastigotes, cells were pulsed with fully competent virus in the absence or presence of amastigotes and virus replication in iDCs cultured alone was evaluated over time. Results depicted in Fig. 3B indicate that the parasite induces a significant increase in *de novo* virus production in iDCs.

**Figure 4:**
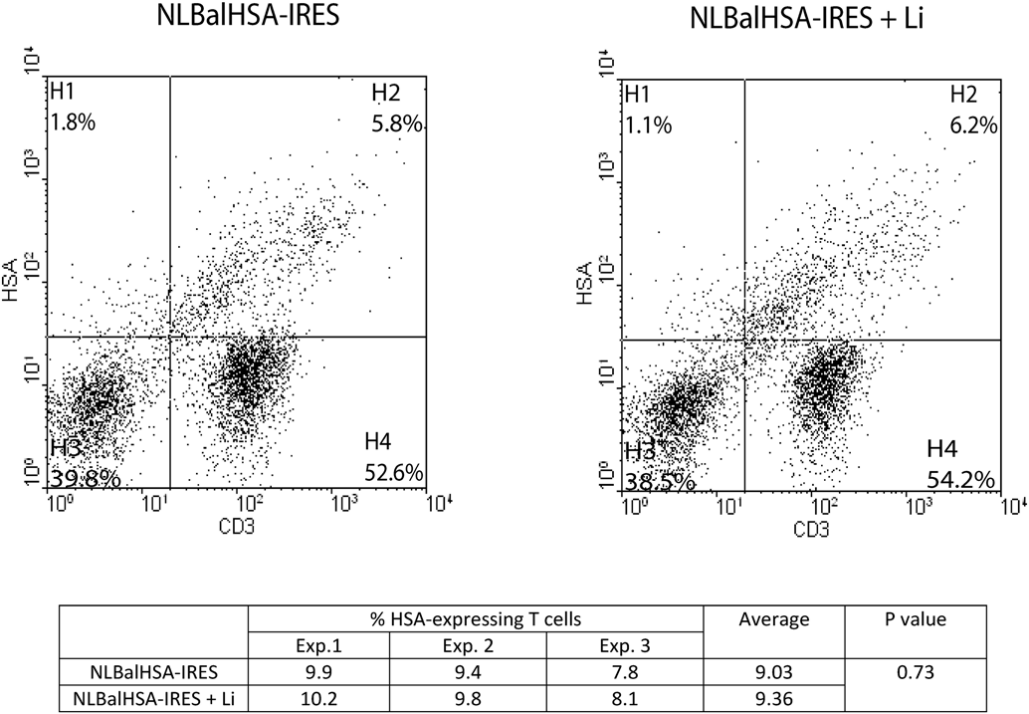
Frequency of virus-infected CD4^+^ T cells in cocultures is not affected by *Leishmania infantum* amastigotes. First, iDCs were pulsed for 60 min with NLBalHSA-IRES virus and either left unexposed or exposed to *Leishmania infantum* amastigotes (Li). Thereafter, iDCs were cocultured with autologous CD4^+^ T cells for 3 days. Finally, cells were stained with anti-CD3 and anti-HSA antibodies before being analyzed by flow cytometry. Results depicted in the right and left panels are representative of a single experiment while the percentage of HSA-expressing cells in the CD3-positive population is shown for three independent experiments (see table).

To provide additional information on how the presence of amastigotes can modulate virus replication in a coculture system made of iDCs and autologous CD4^+^ T cells, we assessed whether the observed increase in virus production is reflected by a corresponding augmentation in the number of CD4^+^ T cells productively infected with HIV-1. To this end, coculture experiments were carried out with NLBalHSA-IRES virus which, upon acute infection of target cells, will produce all viral proteins and also a cell surface reporter molecule (i.e. the murine heat-stable antigen/HSA). Unlike most reporter viruses, fully infectious NLBalHSA-IRES virions have no deletions in the *env*, *vpr* or *nef* genes, and allow for the easy detection of productively infected cells through the surface expression of the HSA molecule [27]. Pulsing iDCs with NLBalHSA-IRES virus followed by a coculture step with autologous CD4^+^ T cells for 3 days resulted in a proportion of CD4^+^ T cells productively infected with HIV-1 ranging from 7.8 to 9.9% as monitored by flow cytometry measuring the relative percentage of HSA and CD3 double-positive cells (Fig. 4). This percentage of virus-infected cells was not affected when amastigotes were present during the pulsing step of iDCs (i.e. percentages of HSA-expressing cells ranging from 8.1% to 10.2%) (mean of 9.03% for cells infected with HIV-1 only compared to 9.36% for cells inoculated with both HIV-1 and *Leishmania*: P = 0.73). This observation suggests that *Leishmania* amastigotes do not affect the number of CD4^+^ T cells which are productively infected with HIV-1, at least at an early time period following initiation of the coculture (i.e. 3 days). Therefore, it can be proposed that *Leishmania* is most likely affecting virus production in the CD4^+^ T cell population by up-regulating virus gene expression.

### *Leishmania*-mediated enhancement in virus replication is due to secretion of IL-6 and TNF-α by iDCs

**Figure 5:**
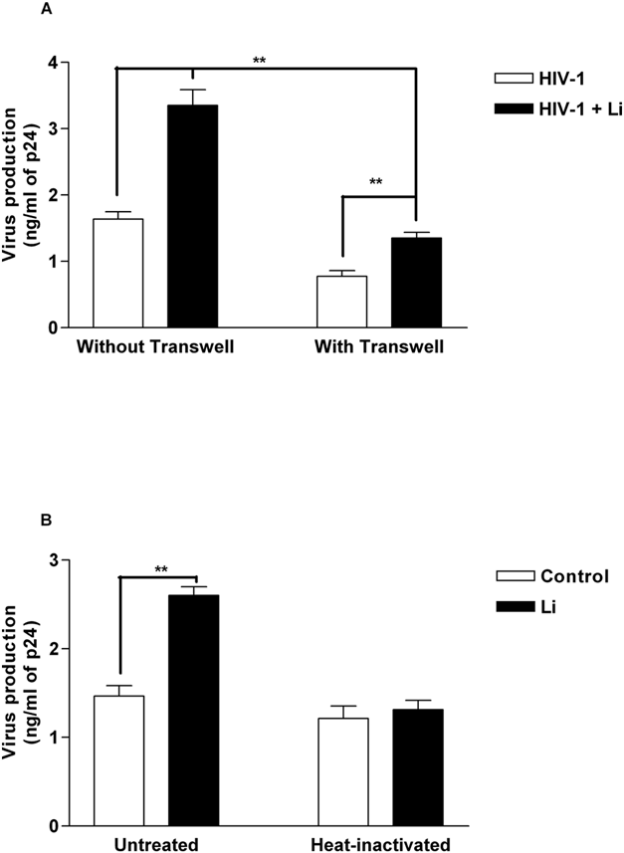
*Leishmania*-dependent increase in virus production seen in cocultured cells is due to a soluble factor(s). (A) First, iDCs were pulsed for 60 min with NL4-3Bal*env* and either left unexposed or exposed to *Leishmania infantum* amastigotes (Li). Next, iDCs were cocultured with autologous CD4^+^ T cells for 3 days either in contact (cell-to-cell contact) or in transwell conditions (no contact). Virus production was estimated by measuring p24 levels in the culture supernatants at 4 days following initiation of the coculture. (B) iDCs were either left unexposed (control) or exposed to *Leishmania infantum* amastigotes (Li) for 24 h. Next, cell-free supernatants were collected and either left untreated or subjected to a heat treatment. Finally, autologous activated CD4^+^ T cells were infected NL4-3Bal*env* and incubated for 3 days with untreated or heat-inactivated cell-free supernatants from iDCs. Virus production was determined by measuring p24 levels in the culture supernatants. Data shown represent the means±SEM of triplicate samples and are representative of five independent experiments. Asterisks denote statistically significant differences from the cells infected with HIV-1 only (**, P<0.01).

To validate that HIV-1 production in CD4^+^ T cells is enhanced upon a coculture with *Leishmania* amastigotes-exposed iDCs, we performed another set of experiments where autologous CD4^+^ T cells were first pulsed with HIV-1 and then cocultured with iDCs either left untreated or inoculated with parasites. Results from Fig. 5A demonstrate that viral replication in CD4^+^ T cells is augmented by a coculture step with *Leishmania*-loaded iDCs. Moreover, we investigated whether a cell-to-cell contact is necessary to achieve the observed phenomenon. To do so, we used permeable cell supports with a membrane pore size of 1 µm, which allows the crossing of virions and soluble factors but not that of cells [29]. When iDCs were separated from CD4^+^ T cells by a permeable membrane, HIV-1 replication decreased in comparison to the contact-favoured cocultured cells (Fig. 5A). However, virus production was still augmented by *Leishmania* amastigotes even when iDCs and CD4^+^ T cells were not allowed to interact with each other. Thus, these results suggest that a soluble factor(s) secreted by *Leishmania*-pulsed DCs drives HIV-1 replication in cocultured cells. Furthermore, when HIV-1-inoculated autologous CD4^+^ T cells were incubated with cell-free supernatants from *Leishmania*-exposed iDCs, an increase in virus replication was seen in CD4^+^ T cells compared to those incubated with cell-free supernatants from unexposed iDCs (Fig. 5B). Interestingly, heat-inactivated supernatants from *Leishmania*-exposed iDCs had no effect on viral replication in CD4^+^ T cells. These observations suggest that the *Leishmania*-directed augmentation in HIV-1 production in cocultures made of iDCs and autologous CD4^+^ T cells is due to a heat-sensitive soluble factor(s) secreted by iDCs.

**Figure 6:**
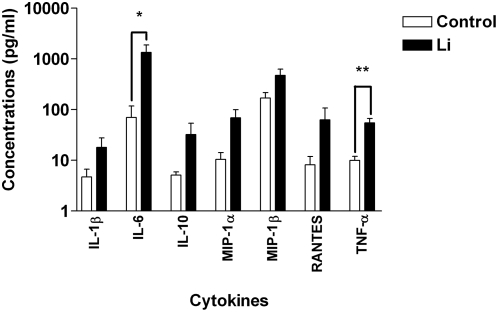
*Leishmania infantum* amastigotes promote secretion of IL-6 and TNF-α. iDCs were either left unexposed (Control) or exposed to *Leishmania infantum* amastigotes (Li) for 24 h. Cell-free supernatants were collected and analyzed with a Bio-Plex assay. The results shown are representative of five separate experiments performed with different donors. Asterisks denote statistically significant differences from the uninfected cells (P<0.05; **, P<0.01).

In order to identify the *Leishmania* amastigote-induced soluble factor(s) responsible for promoting virus replication in both DCs and CD4^+^ T cells, we quantified the levels of several cytokines and chemokines produced by unexposed and *Leishmania*-exposed iDCs using the Multi Analyte Profiling bead array technology from Luminex. Supernatants from *Leishmania*-pulsed iDCs display higher levels of various cytokines and chemokines (i.e. IL-1α, IL-6, IL-10, TNF-α, MIP-1α, MIP-1β and RANTES) than those from untreated iDCs (Fig. 6). However, because of the strong variability between the samples tested, only IL-6 and TNF-α concentrations were increased in a statistically significant manner by *Leishmania infantum* amastigotes.

**Figure 7:**
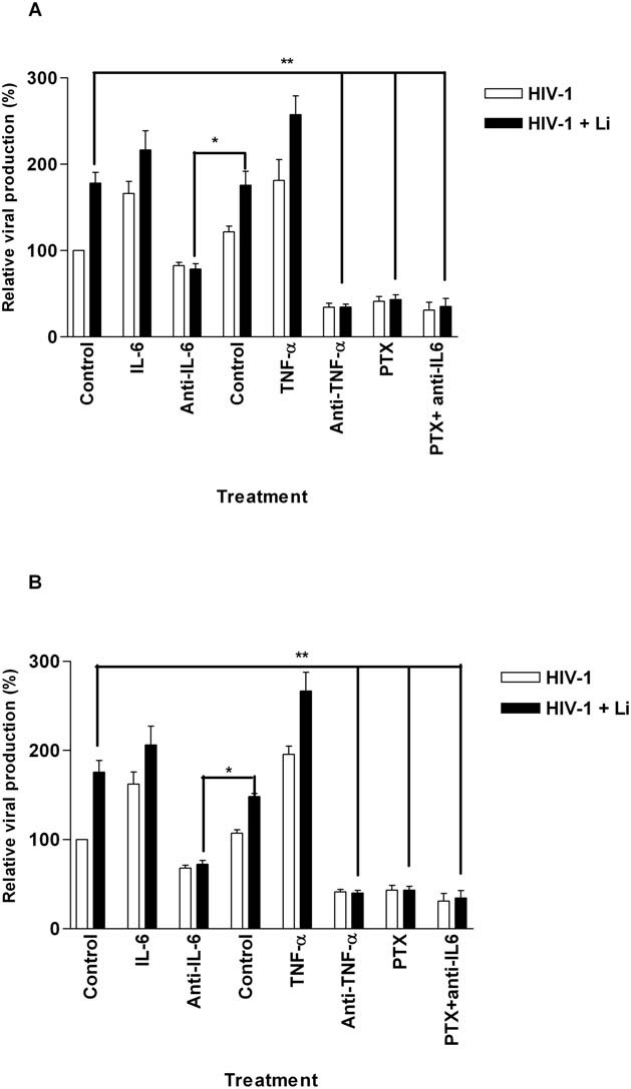
Parasite-mediated enhancement of virus replication in cocultured cells is due to IL-6 and TNF-α. First, iDCs were pulsed for 60 min with NL4-3Bal*env* and either left unexposed or exposed to *Leishmania infantum* amastigotes (Li). Next, iDCs were cocultured with autologous CD4^+^ T cells (A) or cultured alone (B) and either left untreated or treated with the listed reagents (IL-6, 10 U/ml; anti-IL-6, 10 µg/ml; isotype-matched control antibody, 10 µg/ml; TNF-α, 10 ng/ml; anti-TNF-α, 500 ng/ml; and PTX, 100 µM). Virus production was estimated by measuring p24 levels in the culture supernatants either at 4 days following initiation of the coculture (A) or at 6 days post-infection (B). The results shown are representative of four separate experiments performed with different donors and are expressed as relative viral production compared to untreated iDCs. Asterisks denote statistically significant data (*, P<0.05; **, P<0.01).

It has been previously reported that treatment of HIV-1-infected cells with IL-6 and TNF-α results in a significant induction of virus gene expression in a variety of cell systems directly through nuclear translocation of NF-κB [20]–[22],[30]. Therefore, in an attempt to define the relative contribution of these two proinflammatory cytokines to the *Leishmania*-mediated stimulatory effect on virus production, transfer studies were performed with PTX, a phosphodiesterase inhibitor that abolishes TNF-α production [31], and blocking antibodies. Data from Fig. 7A show a direct involvement of both IL-6 and TNF-α in the *Leishmania*-dependent augmentation of HIV-1 replication in cocultured cells. Interestingly, virus production was enhanced upon addition of exogenous IL-6 and TNF-α to cocultured cells, which further implicate both cytokines in the process. Similar patterns were seen when iDCs were cultured alone in absence of autologous CD4^+^ T cells and treated with the listed reagents (Fig. 7B). Altogether, these data indicate that *Leishmania* amastigotes mediate a more important production of IL-6 and TNF-α by iDCs, which in turn stimulates HIV-1 replication in both iDCs and CD4^+^ T cells.

## Discussion

The multifaceted interplay between HIV-1 and *Leishmania* in macrophages has been previously addressed [13],[15]. However, the macrophage is not the only cell type known to harbour both pathogens. Indeed, previous studies have established that DCs are also susceptible to HIV-1 and *Leishmania* infection (i.e. primarily by amastigotes and marginally by promastigotes) [18],[32],[33]. Unfortunately, there is a paucity of data on the consequences of DCs infection with these two microorganisms. A previous study has reported that *Leishmania* reduces HIV-1 capture and transfer by DCs upon pre-incubation with *Leishmania* amastigotes because both pathogens use the cell surface molecule DC-SIGN as a portal of entry in these professional antigen-presenting cells [18]. In the present work, we show that an initial pulsing of DCs with HIV-1 followed by exposure to the amastigote form of the parasite leads to an increased HIV-1 replication in both DCs and CD4^+^ T cells when these two cell subtypes are cocultured together. Interestingly, the promastigote developmental stage of the parasite does not affect virus production under similar experimental procedures. Previous studies have concluded that numerous glycoconjugates that are differentially expressed on the cell surface of the parasite in its promastigote stage (e.g. lipophosphoglycan, leishmanolysin/gp63, low molecular weight glycoinositolphospholipids and a membrane proteophosphoglycan) could be involved in the binding of parasites to target cells [34], but we can conclude that these promastigote-specific molecules have no direct role in the parasite-mediated effect on HIV-1 biology in the DC/CD4^+^ T cell milieu. Moreover, our results are in agreement with other studies showing that amastigotes are internalized by DCs more efficiently than promastigotes [18],[32],[33], therefore validating the hypothesis that engulfment of *Leishmania* parasites is controlled by several factors, such as DC subtypes, *Leishmania* species and parasite stages [16],[17],[35].

It is now well established that DCs can efficiently transmit HIV-1 to neighbouring CD4^+^ T cells through both *trans*- and *cis*-infection pathways. *Trans*-infection mediated by DCs can occur across infectious synapses [8],[9],[11] and via exocytosis of virus-associated exosomes [36], whereas the *cis*-infection route relies on *de novo* virus infection of DCs and long-term transmission of HIV-1 [7],[9],[37],[38]. It is conceivable that both of these mechanisms may coexist and contribute to viral dissemination. In our experimental model, we provide evidence that *Leishmania* amastigotes are affecting HIV-1 replication at different levels in a coculture system consisting of DCs (either iDCs or mDCs) and autologous CD4^+^ T cells. Indeed, we demonstrate that the parasite increases virus production in both DCs and CD4^+^ T cells when these two cell types are cocultured together. Moreover, we report that the percentage of CD4^+^ T cells productively infected by HIV-1 does not seem to be affected by *Leishmania* amastigotes at an early time point after the initiation of the coculture (i.e. 3 days). It can be proposed that the parasite is not modulating the viral transfer process between DCs and CD4^+^ T cells but is instead positively regulating virus gene expression in each cell type. Interestingly, our data indicate that a close contact between *Leishmania*-pulsed DCs and CD4^+^ T cells is not mandatory to stimulate HIV-1 production, therefore suggesting that the observed increase in virus replication is achieved through the secretion of a soluble factor (s).

Elevated plasma protein levels of proinflammatory cytokines and chemokines such as IL-1, IL-6 and TNF-α have been detected in VL patients [39]. Based on this information, it can be proposed that *Leishmania* may modulate HIV-1 replication in our experimental coculture cell system by promoting secretion of proinflammatory cytokines known to amplify virus gene expression. This hypothesis was corroborated by a multiplex fluorescent microfluid immunoassay, which revealed that exposure of DCs to *Leishmania* amastigotes increases secretion of IL-6 and TNF-α. Coculture studies carried out in the presence of neutralizing antibodies specific for IL-6 or TNF-α showed that these two cytokines have a major role in the observed *Leishmania*-induced increase in HIV-1 replication in cocultured cells. A previous study has shown that lipophosphoglycan, a major surface molecule of *Leishmania*, acts as an effective activator of HIV-1 expression in a T lymphoid cell line latently infected with HIV-1 through secretion of TNF-α [40]. It is of interest to note that TNF-α seems to be involved in granuloma formation and the control of parasite growth [41]. For example, anti-TNF-α treatment resulted in the reactivation of VL in patients being treated for arthritis, suggesting a protective role of TNF-α against *Leishmania* infection [42]. On the other hand, when produced at very high levels, TNF-α might have a disease-enhancing effect. For example, one study identified a link between VL and an allelic polymorphism associated with elevated serum levels of TNF-α [43]. High levels of TNF-α promote the generation of IL-10-producing T cells as a homeostatic response to excessive inflammation [44]. It is also well-established that TNF-α significantly increases HIV-1 replication in cells of the macrophage lineage through nuclear translocation of NF-κB [19],[45],[46]. Infection of monocyte-derived macrophages (MDMs) with HIV-1 also induces TNF-α secretion resulting in a positive autocrine loop that enhances virus production [47]. Thus, it can be suggested that the *Leishmania*-mediated release of TNF-α by DCs may function in an autocrine/paracrine manner to induce virus gene expression in cells harboring HIV-1. The same applies to IL-6 as it has been reported to augment HIV-1 replication in MDMs as well as in the latently infected U1 monocytoid cell line. Furthermore, IL-6 has been shown to potentiate TNF-α-induced upregulation of HIV-1 production and induction of NF-κB [21],[22].

Previous studies have shown that PTX, as an effective inhibitor of protein kinase C, protein kinase A and NF-κB, selectively inhibits TNF-α production, as well as HIV-1 transcription and virus production [48],[49]. We show in our study that the parasite-dependent enhancement in HIV-1 production in cocultured cells is no longer seen in presence of PTX. Our observations are in line with a previous report describing that PTX, a methylxanthine derivative that inhibits TNF-α synthesis, diminishes plasma viral load and improves cell-mediated immunity in HIV-1-infected individuals [50]. There is a possibility that PTX might also negatively affect NF-κB signaling in addition to TNF-α production, which could in turn reduce virus gene expression. However, the contribution of TNF-α in the parasite-dependent augmentation of virus production in cocultured cells is confirmed when using blocking anti-TNF-α antibodies. Results presented herein thus show that *Leishmania* amastigotes alter the complex cellular cytokine network and promote secretion of the proinflammatory cytokines IL-6 and TNF-α. Although our data suggest a major role for IL-6 and TNF-α in the *Leishmania*-induced effect on HIV-1 replication in a coculture system constituted of DCs and CD4^+^ T cells, we cannot eliminate the possibility that other soluble factors are involved, either upstream or downstream from them.

HIV-1 and *Leishmania* are the cause of a vast array of immunological disorders. Both infections alter the predominant cellular immune response through complex cytokine-mediated mechanisms that confer susceptibility to both infections. DCs are proposed to have a dominant role in the early events of HIV-1 transmission by transporting the virus from peripheral sites to lymphoid compartments. Moreover, DCs act also as a natural and stable cellular reservoir in the natural course of *Leishmania* infection. The present study illustrates that the effect of *Leishmania* on HIV-1 replication in a coculture system composed of DCs and CD4^+^ T cells largely depends on the developmental stage of the parasite and the chronology of infection of DCs by the two pathogens. We demonstrate that *Leishmania* amastigotes enhance the process of HIV-1 replication by favouring a higher production of the proinflammatory cytokines IL-6 and TNF-α. Data from the current study lead us to speculate about the putative role played by *Leishmania* in the context of a co-infection with HIV-1. The various immunological disturbances caused by *Leishmania* in DCs, a cell population recognized as both a virus and parasite reservoir, can be considered as detrimental to the host by promoting HIV-1 replication and progression of the disease. This assumption is validated by clinical studies showing that leishmaniasis enhances the viral load and reduces life expectancy in HIV-1-infected patients [51]–[53].

In conclusion, this analysis provides novel insights into the complex interconnections between HIV-1 and *Leishmania* and presents unique information that may facilitate the development of more effective therapeutic strategies aimed at controlling disease progression in dually infected individuals.
